# Characterization of Intact *Neo-*Glycoproteins by Hydrophilic Interaction Liquid Chromatography 

**DOI:** 10.3390/molecules19079070

**Published:** 2014-06-30

**Authors:** Alice Pedrali, Sara Tengattini, Giorgio Marrubini, Teodora Bavaro, Petrus Hemström, Gabriella Massolini, Marco Terreni, Caterina Temporini

**Affiliations:** 1 Department of Drug Sciences and Italian Biocatalysis Center, University of Pavia, Via Taramelli, 12, 27100 Pavia, Italy; E-Mails: alice.pedrali@unipv.it (A.P.); sara.tengattini01@ateneopv.it (S.T.); giorgio.marrubini@unipv.it (G.Mr.); teodora.bavaro@unipv.it (T.B.); g.massolini@unipv.it (G.Ms.); marco.terreni@unipv.it (M.T.); 2 Merck SeQuant AB, S-90719 Umeå, Sweden; E-Mail: phemstrom@hotmail.com

**Keywords:** hydrophilic interaction liquid chromatography, intact glycoproteins, semi-synthetic glycosylation monitoring, *neo*-glycoproteins

## Abstract

In this study, an HPLC HILIC-UV method was developed for the analysis of intact *neo*-glycoproteins. During method development the experimental conditions evaluated involved different HILIC columns (TSKgel Amide-80 and ZIC-*p*HILIC), and water-acetonitrile mixtures containing various types of acids and salts. The final selected method was based on a TSKgel Amide-80 column and a mobile phase composed of acetonitrile and water both containing 10 mM HClO_4_. The influence of temperature and sample preparation on the chromatographic performances of the HILIC method was also investigated. The method was applied to the separation of *neo*-glycoproteins prepared starting from the model protein RNase A by chemical conjugation of different glycans. Using the method here reported it was possible to monitor by UV detection the glycosylation reaction and assess the distribution of *neo-*glycoprotein isoforms without laborious sample workup prior to analysis.

## 1. Introduction

Oligosaccharides conjugated to carrier proteins (*neo-*glycoproteins) have been successfully developed as semi-synthetic vaccines; these glycoconjugates are among the safest and most efficacious vaccines available [1,2,3,4,5]. In addition, the concept of synthetic glycoproteins has been extended to the prevention and therapy of several non-infectious diseases such as inflammation, autoimmunity disorders and cancer [6].

The inherent structural complexity of glycosylated proteins necessitates the development of new analytical strategies to characterize these biopharmaceuticals, indeed the structure of therapeutic proteins is critically important for their stability, folding, efficacy and safety. The level of detail and type of information needed (from sequence to conformation) is not the same during production, formulation and quality control stages. In particular, the preparation of structurally well-defined vaccines is required to guarantee the quality of the resulting product (consistent production and immunogenicity) [7]. Therefore, the availability of robust and simple analytical methods for quality control of such glycosylated protein drug products is crucial for the biopharmaceutical industry. 

Characterization of natural glycoproteins in terms of identity, heterogeneity and impurity content has been accomplished using a variety of analytical methods: NMR, MS, CE, HPLC and spectrophotometric methods [8,9]. However, liquid chromatography (LC) coupled to electrospray ionization mass spectrometry (ESI-MS) is the most commonly used approach and has been applied for the analysis of intact glycoproteins, characterization of glycan structures cleaved from proteins and glycopeptides after protein digestion. 

The analysis of the intact proteins is an elegant approach that simplifies sample preparation, but protein heterogeneity can limit resolution [10,11]. The traditional peptide mapping after tryptic digestion of the protein can give a complete picture of the glycosylation sites but is very time-consuming. Analysis of the glycan pool, after enzymatic or chemical cleavage, may provide a detailed picture of the glycan structures but information on the original attachment sites of the glycans is lost [12].

Only the combination of several different approaches can provide the detailed characterization of glycoconjugates that is essential for assessing the influence of cell culture, purification and formulation conditions on protein heterogeneity during the manufacturing process and in order to fulfill the regulatory requirements. There is also a need of simple analytical methods for the quality control and reaction monitoring of synthetic glycosylated proteins. 

In this paper we focused our attention on the development of a simple hydrophilic interaction liquid chromatography (HILIC)-UV method for the analysis of intact glycoproteins to be used for the monitoring of synthetic glycosylation processes. 

LC methods for glycoprotein analysis employ different types of stationary phases, including reversed-phase, graphitized carbon and HILIC. Reversed-phase chromatography is not very suitable for glycoprotein characterization as the resolution is not sufficient to separate chemical modifications to allow their identification at the intact protein level; however a diphenyl column has been used for the resolution of site-specific modifications in IgGs [13]. Graphitized carbon column has demonstrated excellent separation especially for short hydrophilic peptides or oligosaccharides [14] but, as far as we know, this stationary phase has not been used for the analysis of intact glycoproteins. HILIC has gained popularity for glycan and glycopeptide analysis for its ability to retain and separate hydrophilic compounds. Retention is thought to be the result of a complex mixed-mode mechanism, which involves hydrophilic partitioning. Retention for this reason should increase with increasing number of sugars in the glycans/glycopeptides [10,15,16]. In HILIC, acetonitrile/water with volatile buffers are commonly used as mobile phases allowing the interface of the LC system with MS. HILIC has previously been studied for the analysis of intact glycoproteins [17,18].

In this study, two HILIC stationary phases were tested (Amide 80 and ZIC-*p*HILIC) and we demonstrate that the amide-HILIC column gives the best performance in all the evaluated separations. Ribonuclease A (RNase A), intact isoforms of its natural glycosylated variant ribonuclease B (RNase B) and of semi-synthetic *neo*-glycoproteins were used as model proteins for the optimization of the HPLC-UV method. Although mass spectrometric detection provides unparalleled selectivity, our aim is to show that UV can successfully be employed in routine analysis to assess identity and heterogeneity of glycoconjugates during the synthesis phase of the production. In this work, the chromatographic results obtained with the model glycoproteins have been compared with data obtained by MS analyses.

**Scheme 1:**
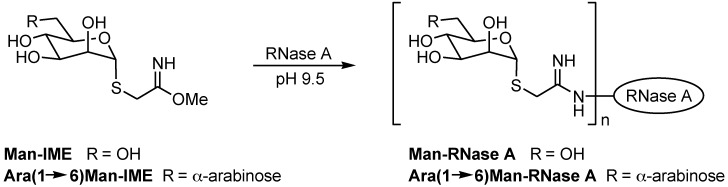
Synthesis of *neo*-glycoproteins by coupling of IME-thioglycoside with RNase A.

The best chromatographic conditions for glycoform separation were then applied to the monitoring of the synthesis of *neo*-glycoconjugates obtained by coupling 2-iminomethoxyethyl-mannose (Man-IME) and arabino-mannose [Ara(1→6)Man-IME] with RNase A (Scheme 1). The developed HILIC-UV method was found suitable for the rapid and easy determination of reaction yields and glycoform distribution.

*Experimental conditions:* 100 mM sodium tetraborate buffer, pH 9.5, 25 °C or 37 °C, molar ratio glycosidic reagent/RNase A 100:1, 24 h.

## 2. Results and Discussion

### 2.1. Optimization of HILIC-UV Method

HILIC is an alternative LC mode for separating polar compounds [19,20]. Recent technological developments in the design of silica-based stationary phases with smaller particles or monoliths have expanded the application of HILIC also to the separation of glycans and glycopeptides [21], while the number of reports on intact glycoprotein analysis is still limited. 

In this work, two different commercial HILIC columns were studied in order to set up an analytical HPLC-UV method able to separate non-glycosylated proteins from the glycosylated counterparts and to resolve the different glycoforms. Particularly, a carbamoyl silica column (TSKgel Amide-80) was chosen since it is widely used for glycopeptide separations [22,23] and was recently described for intact glycoprotein analysis [17,18]. The pore size of this stationary phase is suitable for low and medium molecular weight proteins. A porous polymeric sulfoalkylbetaine column (ZIC-*p*HILIC) was also included. Zwitterionic HILIC columns have been extensively used for the separation of polar compounds including glycopeptides [24] and for selective purification/enrichment of glycosylated peptides [25] but, to our best knowledge, no reports on the separation of intact glycoproteins are published. The polymeric ZIC-*p*HILIC is pH stable and can never exhibit silanol interactions and therefore gives the largest freedom during method development.

The standard proteins selected in this work were RNase A, a 13,681 Da protein, and its glycosylated variant RNase B, which exists in five glycoforms varying for the number of mannose residues (from five to nine) attached to the chitobiose core in the glycan portion.

During the initial method development, column temperature and flow rate were fixed at the convenient values of 25 °C and 0.2 mL/min, respectively. Samples were prepared dissolving the protein/glycoprotein in acetonitrile/water (50:50, v/v) and analyzed injecting a low volume (2 µL) to avoid the deterioration of peak shape due to high percentages of water [26].

Initial experiments were based on existing literature reports [18]. Thus, the TSKgel Amide-80 column and 0.1% (v/v) trifluoroacetic acid (TFA), already described by Zhang and co-workers [18], were used for the separation of the model proteins. Acetonitrile was used throughout as it has lower UV cut-off absorption and viscosity in aqueous mixtures, and because it is known to generate good peak shapes and reproducible results [27,28]. Different gradients with high initial concentration of acetonitrile and increasing concentrations of water were tested to find the conditions providing acceptable sample retention and satisfactory resolution. These aims were achieved applying the following gradient: 68%–58% acetonitrile in 20 min followed by isocratic elution at 58% acetonitrile for 10 min. Under these conditions, RNase A eluted much earlier (*t*_R_ = 13.1 min) than the first eluting glycoform of RNase B (*t*_R_ = 20.2 min) and the five glycosylated isoforms of RNase B could be partially resolved (Rs of 0.8 between the first two glycoforms). In HILIC, the *t*_R_ generally increases with increasing water solubility and polarity of the analyte, and therefore with the length of the glycan chains. 

**Figure 1:**
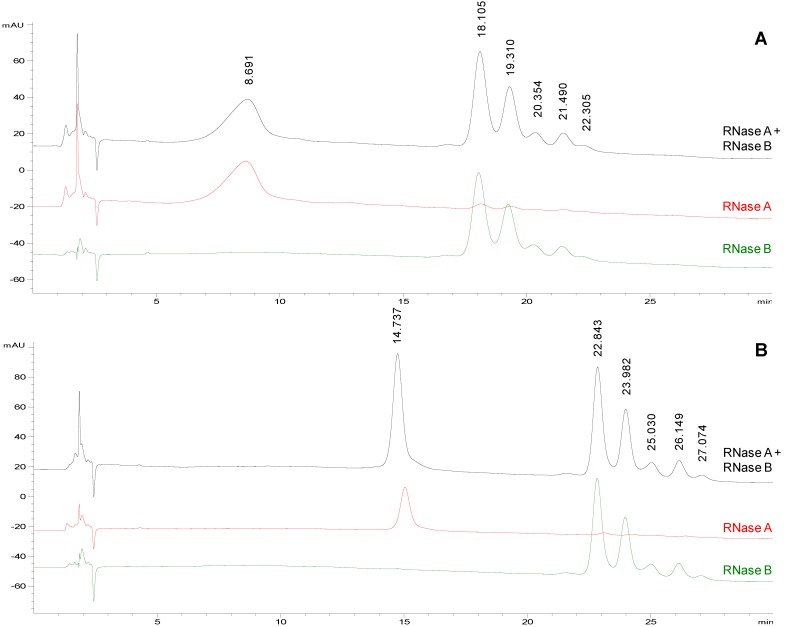
Representative chromatographic profiles for RNase A (red traces), RNase B (green traces) and their equimolar mixture (black traces) on TSKgel Amide-80, eluted at a flow rate of 0.2 mL/min. ^a^ Mobile phases: acetonitrile (solvent A) and water (solvent B) both containing 10 mM HClO_4_. Conditions: from 75 to 65% A in 20 min followed by isocratic elution at 65% A for 10 min. Injection volume: 2 µL. Column temperature: (panel **A**) 25 °C and (panel **B**) 50 °C.

^a^ Samples were prepared in acetonitrile/water (50:50, v/v) at the following concentrations: (panel **A**) 0.5 mg/mL of protein/glycoprotein; (panel **B**) RNase A at 0.25 mg/mL, RNase B and RNase A and B mixture at 0.5 mg/mL.

After these promising results obtained using TFA as mobile phase modifier, a revision of the literature regarding different HPLC modifiers was carried out. The report of Shibue *et al.* [29] suggested that HClO_4_ could provide additional or different selectivity in comparison with TFA, at least in RP-HPLC. Therefore, HClO_4_ was tested in our HILIC separations. The acid was added to mobile phase at concentration close to that of TFA (10 mM). The organic solvent content had to be increased to avoid a too rapid elution of proteins. Figure 1A shows the separation of the model proteins obtained applying the following gradient: 75%–65% acetonitrile in 20 min followed by isocratic elution at 65% acetonitrile for 10 min. With these new chromatographic conditions, higher selectivity and resolution were achieved: RNase A eluted at 8.691 min while RNase B glycoforms started to elute at 18.105 min and were well resolved (Rs of 1.1 between the first two glycoforms) and better separated from the non-glycosylated protein. 

Chromatographic conditions similar to those already described for TSKgel Amide-80 column were then tested on the ZIC-*p*HILIC column. Additionally, a mobile phase containing ammonium acetate (10 mM) was studied. The unsatisfactory results obtained led us to try one additional ZIC-*p*HILIC column with more surface modification and lower surface area prepared specifically for this study. The poor results obtained also with this column lead us to abandon the zwitterionic stationary phase and to direct our efforts on optimizing the chromatographic method using the TSKgel Amide-80 column.

Starting from the chromatographic conditions that led to the best separation of glycoforms on TSKgel Amide 80 column (Figure 1A), the column temperature was increased to 50 °C (Figure 1B) in order to improve the resolution and the peak shapes [30]. As shown in Figure 1 the rise in temperature significantly affected the chromatographic profile prolonging the *t*_R_ (both of protein and glycoprotein isoforms) but also reducing peak widths and improving symmetry. Under these chromatographic conditions, RNase A elutes at a mean *t*_R_ = 14.9 ± 0.5 min (*n* = 5), while the five glycoforms of RNase B (GlcNAc_2_Man_5_ → GlcNAc_2_Man_9_) elute at mean *t*_R_ of 22.7 ± 0.4 min; 23.8 ± 0.4 min; 24.9 ± 0.4 min; 26.0 ± 0.4 min; 26.9 ± 0.4 min, respectively (*n* = 4). The glycoform elution order (from Man5 to Man9) can be assigned with confidence taking into account the ESI-MS spectrum generated by flow injection analysis (FIA) of the same sample as in Figure S1 (Supporting Information). The data reported in literature on commercial RNase B glycoform composition and the retention mechanism exhibited by the TSKgel Amide-80 stationary phase [23,31,32] also supports our peak assignment. Under the selected conditions, also the Rs between the first two glycoforms increases from 1.1 (Figure 1A, 25 °C) to 1.4 (Figure 1B, 50 °C). 

To further improve the glycoform peak shapes and resolution, RNase B analyses were performed dissolving the glycoprotein in a sample diluent containing lower amount of water (acetonitrile/water 90:10, v/v). With this proportion of acetonitrile/water, a small percentage of HClO_4_ (10 mM) had to be added to maintain the glycoprotein in solution. The chromatographic profile obtained was comparable to that already reported for the sample dissolved in acetonitrile/water 50:50 (v/v) (Figures S2 and S3, Supporting Information). This similarity could be explained with a limited influence of the water content in the sample dissolution solvent when a very small volume was injected [26]. In fact, additional experiments performed using an higher injection volume (10 µL) have shown a considerable peak shape distortion due to the presence of an important amount of water (Figures S2 and S3, Supporting Information). Considering these results, we decided to analyze samples prepared in acetonitrile/water 50:50 (v/v) in combination with a low injected volume (2 µL).

In conclusion, the HPLC conditions selected were as follows: mobile phase composed of acetonitrile/water both containing 10 mM HClO_4_, linear gradient from 75% to 65% acetonitrile in 20 min followed by isocratic elution at 65% acetonitrile for 10 min, column temperature at 50 °C, injection volume of 2 μL and flow rate of 0.2 mL/min.

### 2.2. Analysis of Neo-Glycoproteins

The rational design process and the initial synthesis of new glycoconjugate biotherapeutics can gain from analytical methods able to discriminate intact glycoproteins with slight differences in hydrophobicity, as the preliminary studies on glycosylation extent and protein surface reactivity are often carried out using inexpensive short-chain activated polysaccharides. Thus, the optimized analytical method for RNase A and RNase B glycoforms (Section 2.1) was applied to non-natural semi-synthetic glycoconjugates with very small glycan moieties. RNase A was coupled to Man-IME following the reaction depicted in Scheme 1, under the experimental conditions reported in Section 3.2. IME is known to react with the ε-amino group of Lys residues, and the model RNase A (124 amino acids, molecular mass 13,681 Da) contains ten Lys residues. Thus, the condensation of each Man-IME to one Lys ε-amino group results in a mass shift of + 235 Da of the RNase molecular weight, while condensation with Ara(1→6)Man-IME results in a mass shift of + 367 Da per disaccharide unit (Scheme 1). 

After 24 h incubation, the final products were purified and analyzed in parallel by HILIC-UV and FIA-ESI-MS. Glycosylation reactions were performed at different temperatures (reaction 1 at 37 °C and reaction 2 at 25 °C, Table 1) to assess the ability of the method to discriminate differences in the final product composition resulting from the different reaction conditions applied. Data reported in Table 1 are mean values measured from replicate analysis. All the reaction mixtures were purified before the analysis following the procedure described in Section 3.3.2. It should be noted that the full sample preparation process required more than 2 h. The two explored glycosylation conditions resulted in different conjugation conversion percentages (93.1% at 37 °C and 100% at 25 °C) together with different tagging efficiency (moles of mannose units per mole of protein) suggesting that at 25 °C the whole conjugation reaction was more efficient. 

**Table 1:** Man-RNase A glycoform distribution (percentage abundance), conjugation conversion (%C) and Man/protein ratio (mole/mole) as calculated by relative abundance in MS spectra and peak heights in HPLC-UV chromatograms.

#	Analysis (replicates)	n° Man units (%) ± SD								%C ± SD	Man/protein (mole/mole) ± SD
		0	1	2	3	4	5	6	7		
1 ^a^	MS (*n* = 3)	6.9 ± 0.1	20.4 ± 0.3	29.7 ± 0.4	21.7 ± 0.6	14.1 ± 0.4	7.2 ± 0.3	n.d.	n.d.	93.1 ± 0.1	2.37 ± 0.01
	LC-UV (*n* = 3)	6.9 ± 0.1	19.7 ± 0.4	27.0 ± 0.3	24.4 ± 0.2	15.2 ± 0.1	6.8 ± 0.4	n.d.	n.d.	93.1± 0.1	2.42 ± 0.02
2 ^b^	MS (*n* = 3)	n.d	6.9 ± 0.3	14.9 ± 0.4	22.5 ± 0.2	24.8 ± 0.2	16.8 ± 0.3	9.9 ± 0.2	4.1 ± 0.2	100.0	3.76 ± 0.02
	LC-UV (*n* = 4)	n.d.	5.16 ± 0.06	13.58 ± 0.07	22.7 ± 0.2	25.8 ± 0.2	19.4 ± 0.2	9.8 ± 0.2	3.6 ± 0.2	100.0	3.842 ± 0.007

*Experimental conditions:* 100 mM sodium tetraborate buffer, pH 9.5, Man-IME/RNase A 100:1, 24 h, ^a^ 37 °C, ^b^ 25 °C; n.d. not detected.

**Figure 2:**
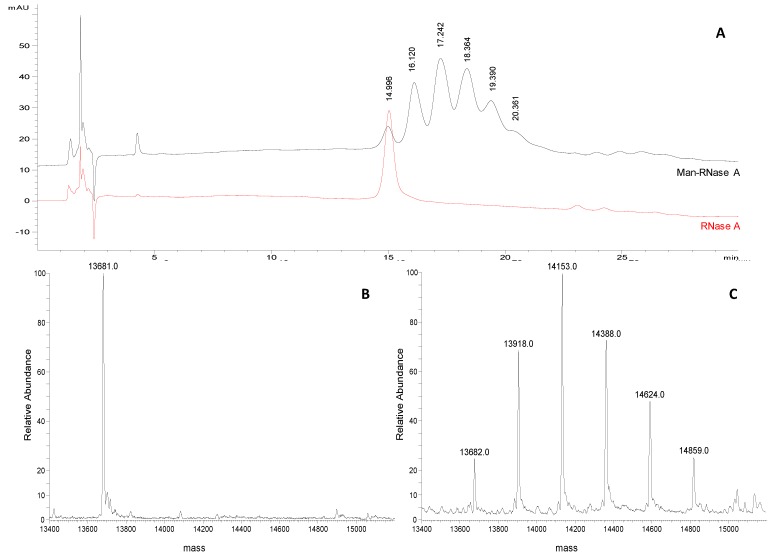
(**A**) Representative chromatograms of Man-RNase A and RNase A (1 mg/mL and 0.25 mg/mL, respectively; acetonitrile/water 50:50, v/v) obtained applying the selected chromatographic conditions (see experimental section). (**B**) Deconvoluted ESI-LTQ-MS spectrum for RNase A (13,681 Da). (**C**) Deconvoluted ESI-LTQ-MS spectrum for Man-RNase A (RNase A 13,681 Da, + 235 Da per mannose unit added).

**Figure 3:**
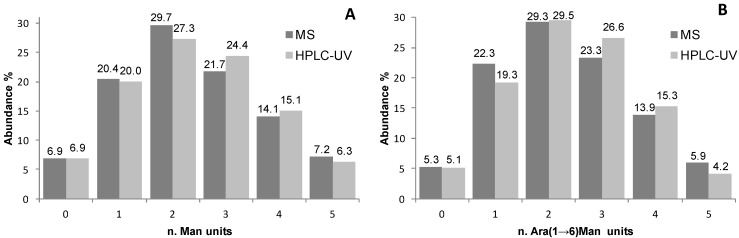
Graphical representation of the glycoform distributions (as abundance percentage) as calculated by relative abundance in MS spectra and peak heights (A) and peak areas (B) in HILIC-UV chromatograms for samples (**A**) Man-RNase A and (**B**) Ara(1→6)Man-RNase A.

A representative chromatographic profile obtained for purified sample 1 (Table 1) is given in Figure 2A. A cluster of six partially resolved peaks can be observed in the chromatogram suggesting the occurrence of several glycoforms in the final product. The first eluting peak (mean *t*_R_ 15.1 ± 0.1 min, *n* = 3) was attributed to unmodified RNase A by comparing the *t*_R_ to that of the pure standard protein (Figure 1B). The following peaks, at 16.2 ± 0.1 min, 17.4 ± 0.1 min, 18.6 ± 0.2 min, 19.6 ± 0.1 min and 20.3 ± 0.1 min, likely represent the individual glycoforms of the resulting mannosylated RNase. Considering their *t*_R_ it might be hypothesised that they correspond to glycoforms carrying from 1 to 5 mannose units, as the mean Δ*t*_R_ between the *neo-*glycoforms is the same as is observed for standard RNase B glycoforms (1.0 min in both) in which the same structural change, *i.e.*, one mannose unit, occurs. This hypothesis was corroborated using FIA-ESI-MS data. The ESI-MS spectrum of the same sample (Figure 2C) resulted in an overlapping *neo-*glycoprotein profile composition. The presence of unreacted RNase A was confirmed by the presence of the 13,682 Da signal (Figure 2B,C), as well as the consecutive glycoforms bearing from 1 to 5 mannose units (+ 235 Da per IME-activated monosaccharide unit). The same correspondence between both semi-quantitative methods was seen in all the glycosylation experiments monitored (sample 1, and sample 2 in Table 1) (Figure 3A). 

**Figure 4:**
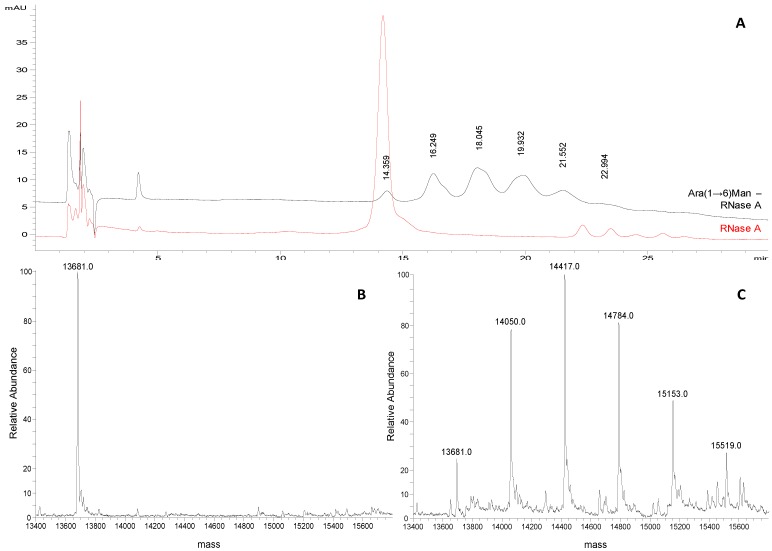
(**A**) Representative chromatograms of Ara(1→6)Man-RNase A and RNase A (1 mg/mL and 0.25 mg/mL, respectively; acetonitrile/water 50:50, v/v) obtained applying the selected chromatographic conditions (see experimental section). (**B**) Deconvoluted ESI-LTQ-MS spectrum for RNase A (13,681 Da). (**C**) Deconvoluted ESI-LTQ-MS spectrum for Ara(1→6)Man-RNase A (RNase A 13,681 Da + 367 Da per Ara(1→6)Man unit added).

Ara(1→6)Man-IME glycoconjugates obtained with the same reaction protocol were also analyzed. A representative chromatographic profile is given in Figure 4A, where the non-modified protein (*t*_R_ 14.359 min) and the five glycoderivatives elute showing almost baseline separation. Four replicate analysis were carried out and the consecutive glycoforms elute at mean *t*_R_of 16.1 ± 0.3 min; 17.9 ± 0.3 min; 19.8 ± 0.3 min; 21.4 ± 0.2 min; 22.9 ± 0.2 min, respectively. From the data obtained, two main observations were made: first, the mean Δ*t*_R_ between the consecutive glycoforms increases from 1.0 min to 1.75 min compared to the mannose derivatives. This is in accordance with the different length of the attached glycan, proving the HILIC mechanism. Secondly, the peak shapes of the Ara(1→6)Man derivatives are neither symmetric nor regular. This problem might be related to glycosylation site heterogeneity, that is more evident when the saccharide chain length increases. 

For this reason in the quantitative measurements peak areas instead of peak heights were compared to the relative abundances of the different products calculated from the ESI-MS of the same samples (Figures 3B and 4C). Also in this case, the same semi-quantitative data (Figure 3B) and structural information (Figure 4A–C) can be derived from both techniques, cross validating each-other. 

**Figure 5:**
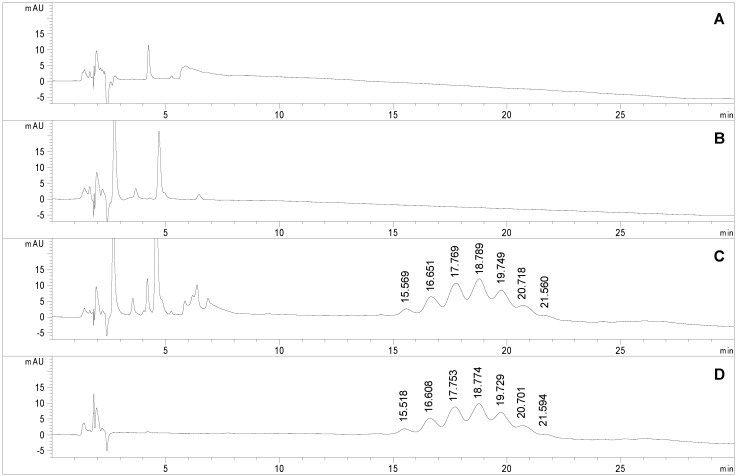
Chromatograms obtained applying the selected conditions (see experimental section) to the analyses of (**A**) sodium tetraborate buffer (100 mM, brought to pH 6 with HCl and diluted 1:2 with acetonitrile), (**B**) Man-IME (1 mg/mL in acetonitrile/water 50:50, v/v); (**C**) non-purified and (**D**) purified Man-RNase A (0.4 mg/mL in acetonitrile/water 50:50, v/v).

At the present state of the art in glycoprotein analysis, one of the main bottlenecks limiting analysis throughput is the sample preparation step. In the case of protein glycosylation, as already stated, a 2 h sample clean-up is required to remove reagents and salts after the coupling reaction. This step is mandatory prior to any FIA-ESI-MS analysis due to the presence of non volatile salts, while the HILIC-UV method could avoid the time consuming purification step. A study was carried out to assess the chromatographic profile of each component of the coupling reaction mixture to verify method selectivity. Figure 5 reports the chromatographic profiles obtained upon injection of the reaction buffer (trace A), the Man-IME reagent (trace B) and the final reaction product (namely sample 2, Table 1) before (trace C) and after (trace D) the purification protocol. All solvents and chemical reagents elute in the first part of the chromatogram without interfering with the macromolecules. No significant effect on the *t*_R_ of the glycoforms occurred, the Δ*t*_R_ between consecutive glycoforms was estimated to 1.00 min in the non purified sample, and 1.01 min in the purified one. This demonstrates that the presence of high amounts of Man-IME reagent (trace C) does not affect the selectivity of the stationary phase.

Also the semi-quantitative data on crude samples were highly consistent with those obtained with the purified samples (sample 2, Table 1): indeed, glycoform composition of the raw sample (trace C) was estimated as 5.1% (Man1), 13.6% (Man2), 22.8% (Man3), 26.1% (Man4), 19.1% (Man5), 9.5% (Man6), 3.7% (Man7).

**Figure 6:**
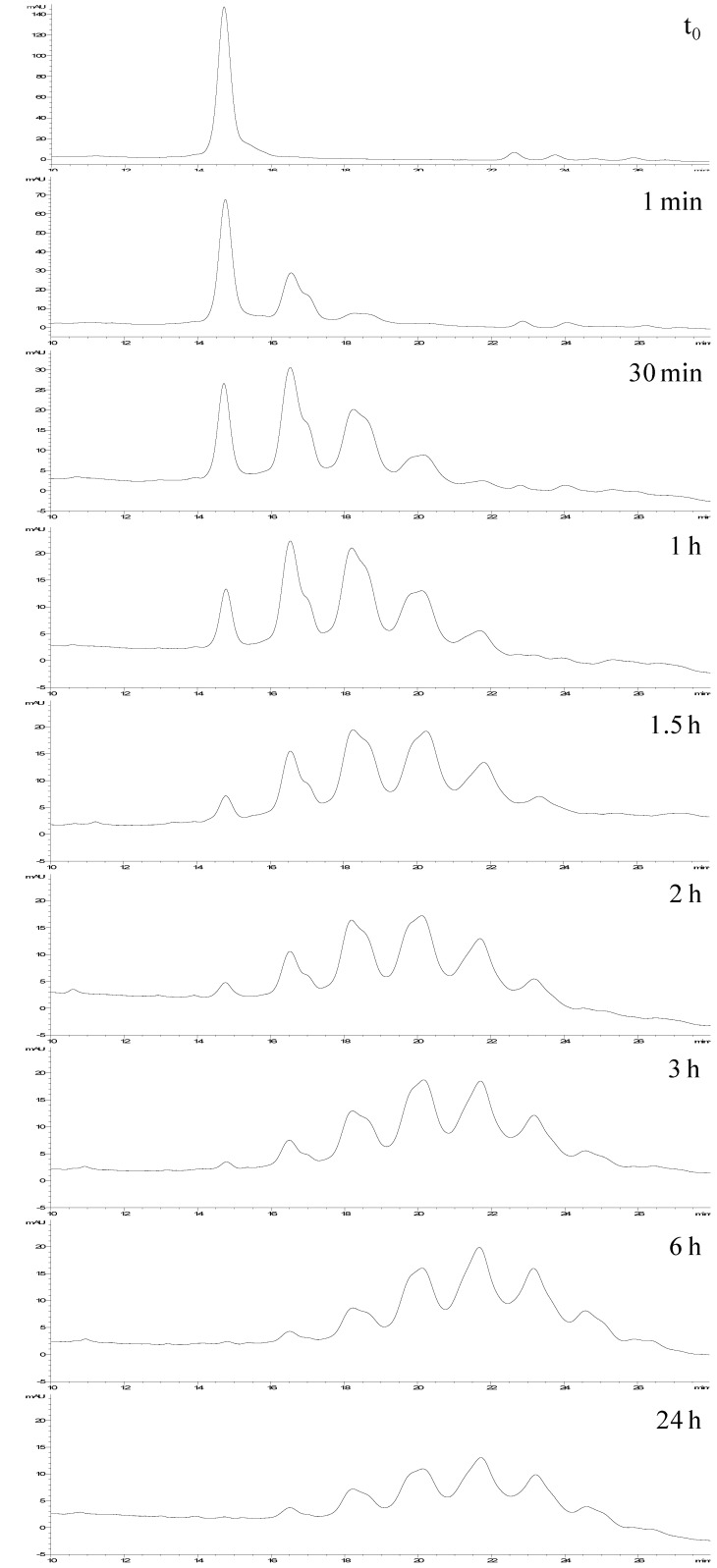
Monitoring of the synthesis of *neo*-glycoconjugates by coupling Ara(1→6)Man-IME with RNase A.

### 2.3. Application of the HILIC-UV Method to Conjugation Reaction Monitoring

When a *neo-*glycoprotein is prepared *via* a semi-synthetic route reaction monitoring is by measuring the consumption of the reagents, in this case the non modified protein, from which the reaction yield can be assessed. Additionally, the capability to separate and quantify the individual glycoderivatives produced allows much better control over the actual conversion to the desired product. The analysis of intact protein/*neo-*glycoproteins at the end or during the coupling reaction represents a rapid alternative to peptide mapping. In addition, the omission of sample preparation step guarantees little sample consumption and high analysis throughput.

For these reasons the selected HILIC-UV method was applied to investigate the kinetics of the conjugation process by monitoring the reaction of RNase A with the disaccharide Ara(1→6)Man-IME.

The reaction was carried out according to protocol (Section 3.2) at 25 °C for 24 h and samples were collected at scheduled times (1 min, every 30 min for the first 3 h, at 4 h, 6 h, 8 h and the end of the reaction). Each sample was brought to pH 5–6 to stop the glycosylation reaction and injected in the HPLC system without purification (as described in Section 3.3.2). Figure 6 shows representative chromatograms of this reaction monitoring. At each analyzed time, the HPLC-UV analysis allowed us to determinate the conjugation conversion (by the % of non-reacted protein) and the number as well as distribution of the generated glycoforms. 

Additionally, it was interesting to prove that, under these experimental conditions, after 6 h the reaction is complete, resulting in a stable product with well-defined composition (see time 6 h *vs.* 24 h). Parallel to LC-UV analysis, ESI-MS direct infusion analyses of the same samples were carried out, and showed that the relative abundances of the detected species were fully in agreement with the HILIC-UV results (Table 2). 

These results demonstrate that this HILIC-UV method represents an option easier and quicker than FIA-ESI-MS for accurate monitoring of the chemical coupling of glycans with the selected protein.

In an attempt to elucidate the origin of the asymmetric peak shape for the Ara(1→6)Man *neo-*glycoproteins a sample from *t* = 1 min was analysed, at this time the reaction mixture contained almost only RNase A and the species with one incorporated Ara(1→6)Man residue. This sample was purified and digested with chymotrypsin and the glycopeptides were analysed by the HPLC-MS*^n^* method reported in Section 3.7. Representative MS/MS and MS^3^ spectra of two selected glycopeptides are reported in Figure S4 (Supporting Information). 

**Table 2:** Ara(1→6)Man-RNase A glycoform distribution (percentage abundance), conjugation conversion (%C) and Ara(1→6)Man/protein ratio (mole/mole) as calculated by relative abundance in MS spectrum and peak areas in HILIC-UV chromatogram.

Reaction Time	Analysis	N. Ara(1→6)Man Units (%)								%C	Ara(1→6)Man/Protein Ratio (mol/mol)
		0	1	2	3	4	5	6	7		
0 min	MS	100.0	n.d.	n.d.	n.d.	n.d.	n.d.	n.d.	n.d.	0.0	0.0
	HPLC-UV	100.0	n.d.	n.d.	n.d.	n.d.	n.d.	n.d.	n.d.	0.0	0.0
1 min	MS	50.5	37.5	12.0	n.d.	n.d.	n.d.	n.d.	n.d.	49.5	0.6
	HPLC-UV	53.3	36.6	10.0	n.d.	n.d.	n.d.	n.d.	n.d.	46.7	0.6
30 min	MS	21.2	38.0	27.3	13.6	n.d.	n.d.	n.d.	n.d.	78.8	1.3
	HPLC-UV	18.8	37.8	30.5	12.8	n.d.	n.d.	n.d.	n.d.	81.2	1.4
1 h	MS	12.7	29.8	28.6	19.6	9.3	n.d.	n.d.	n.d.	87.3	1.8
	HPLC-UV	8.7	26.7	34.6	22.1	7.9	n.d.	n.d.	n.d.	91.3	1.9
1.5 h	MS	5.8	18.3	26.3	25.6	16.3	7.7	n.d.	n.d.	94.2	2.5
	HPLC-UV	3.9	17.0	28.2	28.6	16.9	5.4	n.d.	n.d.	96.1	2.5
2 h	MS	4.2	13.7	24.2	27.2	20.4	10.3	n.d.	n.d.	95.8	2.8
	HPLC-UV	2.6	12.1	26.4	29.7	22.1	7.1	n.d.	n.d.	97.4	2.8
3 h	MS	3.7	8.4	18.6	24.8	22.5	14.0	7.9	n.d.	96.3	3.3
	HPLC-UV	1.3	6.7	17.3	27.5	25.6	16.3	5.4	n.d.	98.7	3.4
6 h	MS	n.d.	4.9	13.3	21.2	24.1	19.5	12.8	4.3	100.0	4.0
	HPLC-UV	n.d.	2.6	10.6	22.2	27.9	22.8	10.5	3.4	100.0	4.0
24 h	MS	n.d.	5.2	14.0	21.7	23.6	19.0	12.6	3.9	100.0	3.9
	HPLC-UV	n.d.	3.6	12.7	22.7	27.9	20.6	8.9	3.5	100.0	3.9

*Experimental conditions:* 100 mM sodium tetraborate buffer, pH 9.5, Ara(1→6)Man-IME/RNase A 100:1, 25 °C; n.d. not detected.

**Table 3:** List of glycopeptides identified by LC-ESI-MS*^n^* from the chymotryptic digestion of sample *t* = 1 min in the monitoring of the synthesis of Ara(1→6)Man-RNase A.

t_R_ (min)	*m/z*	Charge State	Theor. Mass (Da)	Exper. Mass (Da)	Position	Glycosylation Site	Area %
5.96	558.64	2	1115.26	1115.28	M_30_-L_35_	K_31_	6.78
6.10	617.17	2	1232.34	1232.34	K_1_-F_8_	K_1_ or K_7_	50.03
	1233.71	1		1232.71			
6.44	493.08	2	984.06	984.16	K_31_-L_35_	K_31_	7.09
6.89	788.08	3	2362.50	2361.24	S_80_-Y_97_	K_91_	26.23
	1181.75	2		2361.50			
8.35	675.82	3	2025.18	2024.46	S_59_-Y_73_	K_61_ or K_66_	9.88
	1012.63	2		2023.26			

The obtained data revealed the presence in the sample of at least four glycoforms with different conjugation sites, namely Lys 1 or 7 (50.0%), Lys 31 (13.9%), Lys 61 or 66 (9.9%) and Lys 91 (26.2%) (Table 3). This experimental evidence supports the hypothesis that the non-Gaussian peak shape is related to the co-elution of different *neo-*glycoprotein isomers.

## 3. Experimental

### 3.1. Reagents and Chemicals

α-Chymotrypsin, dithiothreitol (DTT), RNase A and RNase B from bovine pancreas were purchased from Sigma-Aldrich (Milan, Italy) and were used without further purification. Potassium dihydrogen phosphate and ammonium acetate were from Merck (Darmstadt, Germany) and of analytical grade purity. Sodium tetraborate (99%), trifluoroacetic acid (>99%), HClO_4_ (70%), and ammonium bicarbonate (99%) were from Sigma-Aldrich. Formic acid (99%), hydrochloric acid (37%) and ortophosphoric acid (85%) were purchased from Carlo Erba Reagenti (Milan, Italy). Water was obtained from a Direct-QTM system Millipore (Millipore, Milan, Italy). Acetonitrile gradient grade for HPLC CHROMASOLV^®^ (*>*99.9%) was purchased from Sigma-Aldrich. 2-Iminomethoxyethyl (IME) thioglycosides (mannose-IME and arabinose(1→6)mannose-IME) were prepared according to the previously reported methods [33,34,35].

### 3.2. Glycosylation Procedure

According to the glycosylation protocol described by Davis [35], the reaction was carried out in sodium tetraborate buffer, 100 mM, pH 9.5. RNase A was dissolved in the buffer to reach a final concentration of 1.7 mg/mL and then the solution was mixed with IME-glycoside to a glycoside/protein molar ratio of 100/1. The reaction mixture was vortexed for 1 min and incubated for 24 h at different temperatures (25 °C and 37 °C) under continuous stirring. The reaction was stopped by addition of HCl (37%) to pH 5–6.

### 3.3. Sample Preparation

#### 3.3.1. Standard Solutions

Standard stock solutions of RNase A and RNase B were prepared in pure water at a concentration of 2 mg/mL and then diluted with acetonitrile/water mixtures (different percentages) to obtain working solutions at 0.25–1 mg/mL.

#### 3.3.2. *Neo-*glycoprotein Solutions

*Neo-*glycoproteins, prepared as described in Section 3.2, were purified in order to remove reagents and salts. The reaction mixture was submitted to seven 20 min steps of ultrafiltration at 13,000 g and 4 °C using centrifuge 5804-R (Eppendorf s.r.l., Milan, Italy) and Millipore’s Amicon^®^ Ultra filters with Nominal Molecular Weight Limit (NMWL) of 3 kDa and load capacity of 500 µL. Deionized water was used as washing solution. After that, the *neo-*glycoproteins were recovered by turning filter upside down in clean tube and spinning for 2 min at 1,000 g at 4 °C. Finally, samples were diluted to obtain the desired solutions. MS analyses were performed on 0.3 mg/mL solutions prepared with acetonitrile/water (50:50, v/v) and 0.05% TFA, while HPLC chromatograms were recorded analyzing samples in acetonitrile/water (50:50, v/v) at concentration levels in the range of 0.4–1 mg/mL. Non-purified *neo-*glycoproteins were analyzed in the reaction mixture previously brought to pH 5–6 with HCl (37%) and diluted to reach final concentration levels 0.4 mg/mL or 0.8 mg/mL in acetonitrile/water (50:50, v/v).

### 3.4. Instrumentation and HILIC Chromatographic Conditions

Chromatographic separations of intact proteins were performed on an Agilent HPLC series 1200 system, equipped with mobile-phase online degasser, quaternary pump, autosampler, column thermostated compartment, and diode array detector. For data acquisition and analysis, the ChemStation software version Rev. B.04.01 was used in a Microsoft Windows XP environment.

The HILIC columns studied were: TSKgel Amide-80 (2 × 150 mm, 3 μm, 80 Å) from Tosoh Bioscience (Montgomeryville, PA, USA) and Merck SeQuant^®^ ZIC-*p*HILIC (2.1 × 150 mm and 4.6 × 50 mm, 5 μm, Umeå, Sweden).

Standard protein and *neo-*glycoprotein separations were performed using a TSKgel Amide-80 (2 × 150 mm, 3 μm, 80 Å) column. The mobile phase was composed of acetonitrile (solvent A) and water (solvent B) both containing 10 mM HClO_4_. Chromatographic conditions were: 20 min linear gradient from 75% to 65% solvent A followed by an isocratic elution at 65% solvent A for 10 min. The column temperature was maintained at 50 °C, the injection volume was 2 μL and elution was carried out at constant flow of 0.2 mL/min. In all experiments proteins were detected photometrically at 210 nm.

### 3.5. ESI-MS Analysis of Intact Glycoproteins

Intact MS experiments were carried out on a Linear Trap Quadrupole (LTQ) mass spectrometer with electro-spray ionization (ESI) source (Thermo Finnigan, San Jose, CA, USA). The system was controlled by Xcalibur software 1.4 (Thermo Finnigan). 

The purified samples were directly introduced into the mass spectrometer with a 100 μL syringe moved at 10 µL/min by a syringe pump (Thermo Fisher Scientific, San Jose, CA, USA). Full scan intact MS experiments were carried out under the following instrumental conditions: positive ion mode; mass range, 900–2000 *m/z*; source voltage, 4.5 kV; capillary voltage, 35 V; sheat gas, 15 (arbitrary units); auxiliary gas, 2 (arbitrary units); capillary temperature, 220 °C; tube lens voltage, 140 V.

Multiple-charged proteins ion signals were deconvoluted by using Bioworks Browser (Thermo Electron, revision 3.1) and the percentage abundance of different glycoforms calculated by the relative abundance of corresponding peaks in the deconvoluted spectra.

### 3.6. Protein Digestion Protocol

For the chymotryptic digestion, 50 µL of 100 µM protein solution in water was added with 45 µL of 100 mM ammonium bicarbonate, pH 8.5, and 5 µL of DTT solution (100 mM in 100 mM ammonium bicarbonate, pH 8.5). The solution was first heated at 60 °C for 30 min for disulfide bridge reduction, and then addition of chymotrypsin to a final protein/enzyme ratio of 100:1. The solution was incubated overnight at 37 °C under continuous stirring. The reaction was stopped by adding 2.5% TFA.

### 3.7. HPLC-MS Analysis of Glycopeptides

The digestion mixture was analyzed according to a method previously described by Temporini *et al.* [31] with some modification. Briefly, glycopeptides were *on-line* extracted on a porous graphitized carbon trap column Hypersil Hypercarb (50 × 4.6 mm), purchased from Alltech Associates (Deerfield, IL, USA) applying the following conditions: 10 min of desorption from the trap column with 80% solvent A (acetonitrile + 0.05% TFA) and 20% solvent B (water + 0.05% TFA) at 100 µL/min. Glycopeptide separation was performed on a Dionex Ultimate 3000 HPLC system (Sunnyvale, CA, USA) controlled by Chromeleon software (version 6.8) using a TSK-gel Amide-80 column (150 × 2 mm, 3 µm, 80 Å) purchased from Tosoh Bioscience LLC (Montgomeryville, PA, USA). The gradient of HPLC analysis was from 30% to 57% B in 11 min with a flow rate of 200 µL/min. The analytes were revealed by an ESI-LTQ-MS controlled by X-calibur software 1.4 (Thermo Finnigan). Mass spectra were generated in positive ion mode under the following instrumental conditions: source voltage, 4.0 kV; capillary voltage, 46 V; sheath gas flow, 40 (arbitrary units); auxiliary gas flow, 10 (arbitrary units); sweep gas flow, 1 (arbitrary units); capillary temperature, 250 °C; and tube lens voltage, −105 V. MS^2^ and MS^3^ spectra were obtained by CID.

Glycopeptides were identified on the basis of MS^2^ and MS^3^ spectra by Bioworks Browser (Thermo Electron, revision 3.1) considering the glycan moiety as differential modifications of lysine residues. To avoid false positive the spectra of all species recognized as glycopeptides were manually evaluated.

## 4. Conclusions

In this work a rapid HILIC-HPLC-UV method was developed for the separation of intact proteins and their glycoconjugates. It was demonstrated that the HILIC amide column is able to discriminate the different glycoforms on the basis of their saccharide composition. The method was applied to investigate the kinetics of the conjugation process by monitoring the synthesis of *neo*-glycoproteins. These experiments proved that the developed HILIC method is a valuable tool to rapidly monitor the reaction degree and to assess the number and relative abundance of produced glycoforms. Interestingly, the semi-quantitative output drawn by this simple LC-UV method is highly consistent with that obtained with FIA-ESI-MS methods, while requiring no sample preparation, a remarkable advantage in terms of throughput and cost.

## Supplementary Materials

Supplementary materials can be accessed at: http://www.mdpi.com/1420-3049/19/7/9070/s1.
