# Supplementary material for: Characterization of Intact Neo-Glycoproteins by Hydrophilic Interaction Liquid Chromatography
Source: Molecules. 2014 Jun 30;19(7):9070–88. doi: 10.3390/molecules19079070 (PMC6271321; doi:10.3390/molecules19079070)

Supporting Information

Table S1. Details on RNAse A – Man reactions samples and replicates reported in Table 1 (Sample 1 at T = 37 ℃ and Sample 2 at 25 ℃).

Sample 37 ℃	n °Man Units (%)									
	0	1	2	3	4	5	6	7	%C	Man/Protein Ratio (mole/mole)
**1**	4.2	15.1	27.7	28.4	18.6	6.0	n.d.	n.d.	95.8	2.6
**2**	5.4	16.5	27.6	28.6	16.2	5.7	n.d.	n.d.	94.6	2.5
**3**	6.9	20.4	29.7	21.7	14.1	7.2	n.d.	n.d.	93.1	2.4
	5.5 ±1.3	17.3 ±2.7	28.3 ±1.2	26.2 ±3.9	16.3 ±2.3	6.3 ±0.8	-	-	94.5 ±1.3	2.5±0.1

Sample 25 ℃	n °Man Units (%)									
	0	1	2	3	4	5	6	7	%C	Man/Protein Ratio (mole/mole)
**1**	n.d.	2.0	9.3	16.2	24.8	22.7	15.6	9.5	100.0	4.4
**2**	n.d	2.7	9.9	16.7	24.5	22.4	15.3	8.5	100.0	4.3
**3**	n.d.	4.5	11.4	19.2	24.3	19.4	13.6	7.5	100.0	4.1
**4**	n.d	6.9	14.9	22.5	24.8	16.8	9.9	4.1	100.0	3.8
	-	4.0 ±2.2	11.4 ±2.5	18.6 ±2.9	24.6 ±0.3	20.3 ±2.8	13.6 ±2.6	7.4 ±2.3	100.0	4.2 ±0.3

Figure S1. ESI-MS deconvoluted spectrum, identity and relative abundance of commercial RNase B glycoforms. Sample conc. (0.3 mg/mL) in acetonitrile/water (50:50, v/v) with 0.05% TFA, FIA analysis carried out under conditions reported in Section 3.5.

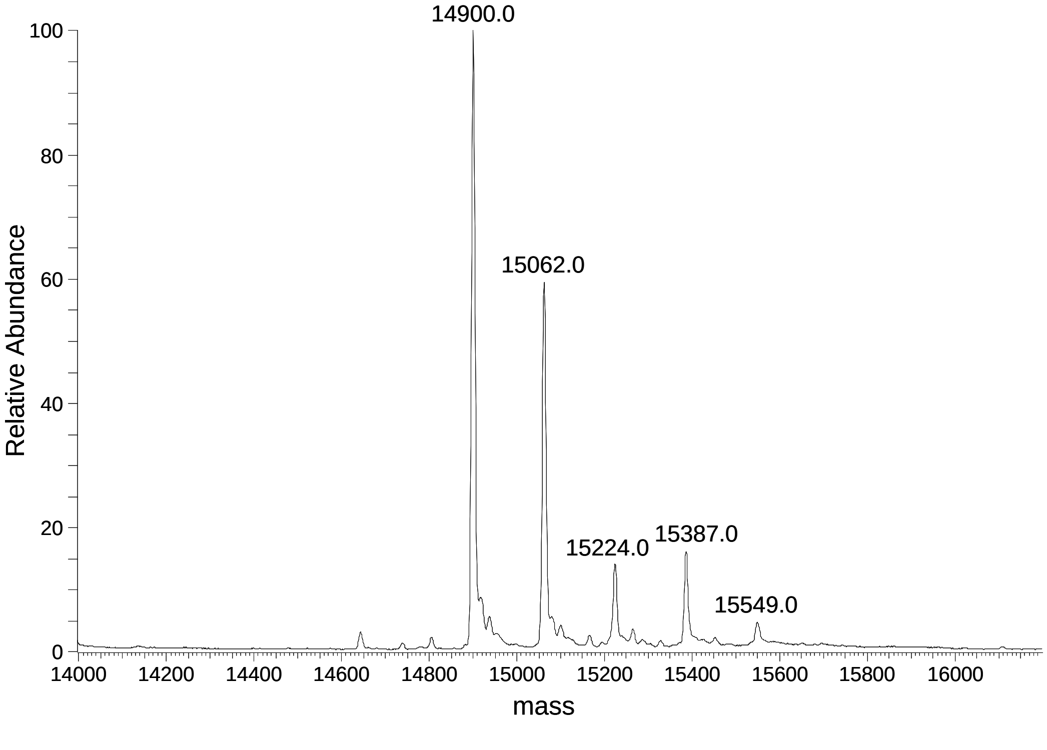

Figure S2. Analyses of RNase B 0.5 mg/mL dissolved in acetonitrile/water (90:10, v/v) in presence of $HClO_4$ 10 mM and analyzed at different injection volumes (2, 5 and 10 μL).

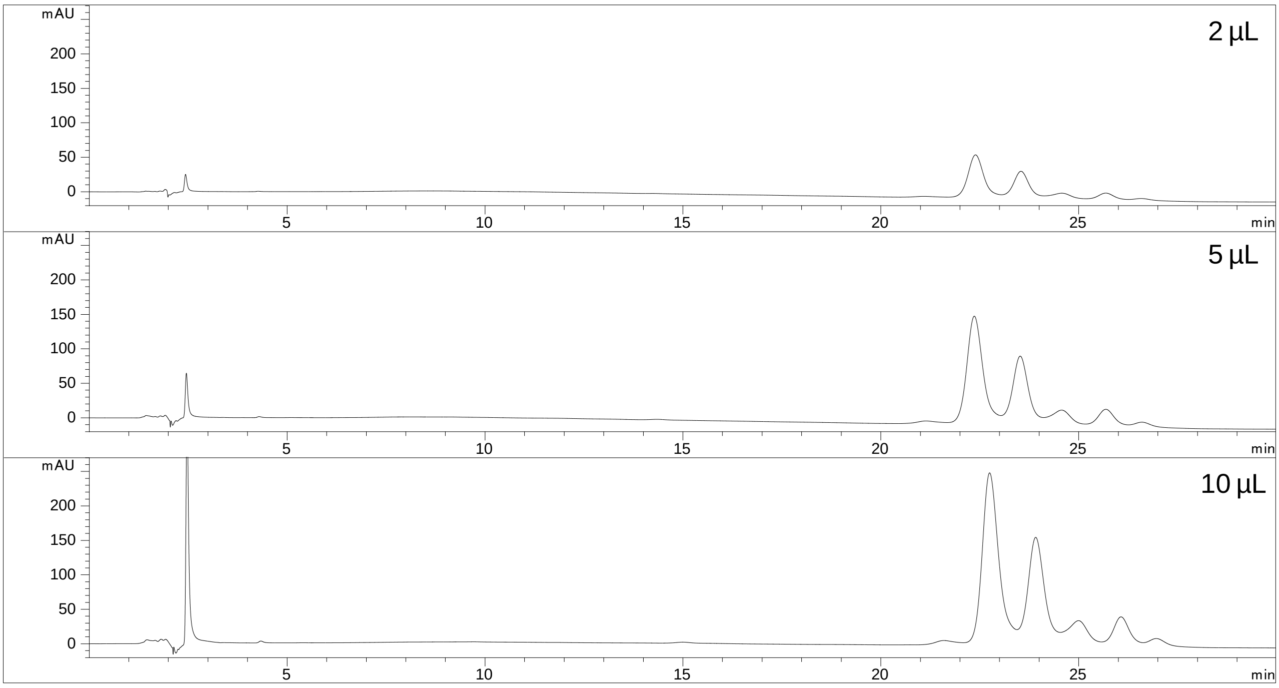

	$GlcNAc_2Man_5$	$GlcNAc_2Man_6$	$GlcNAc_2Man_7$	$GlcNAc_2Man_8$	$GlcNAc_2Man_9$
MW (Da)	14,900.0	15,062.0	15,224.0	15,387.0	15,549.0
Intensity	100	59.5	14	16	5
Abundance %	52.2	30.9	6.9	7.9	2.1

Figure S3. Analyses of RNase B 0.5 mg/mL dissolved in acetonitrile/water (50:50, v/v) in presence of $HClO_4$ 10 mM and analyzed at different injection volumes (2, 5 and 10 μL).

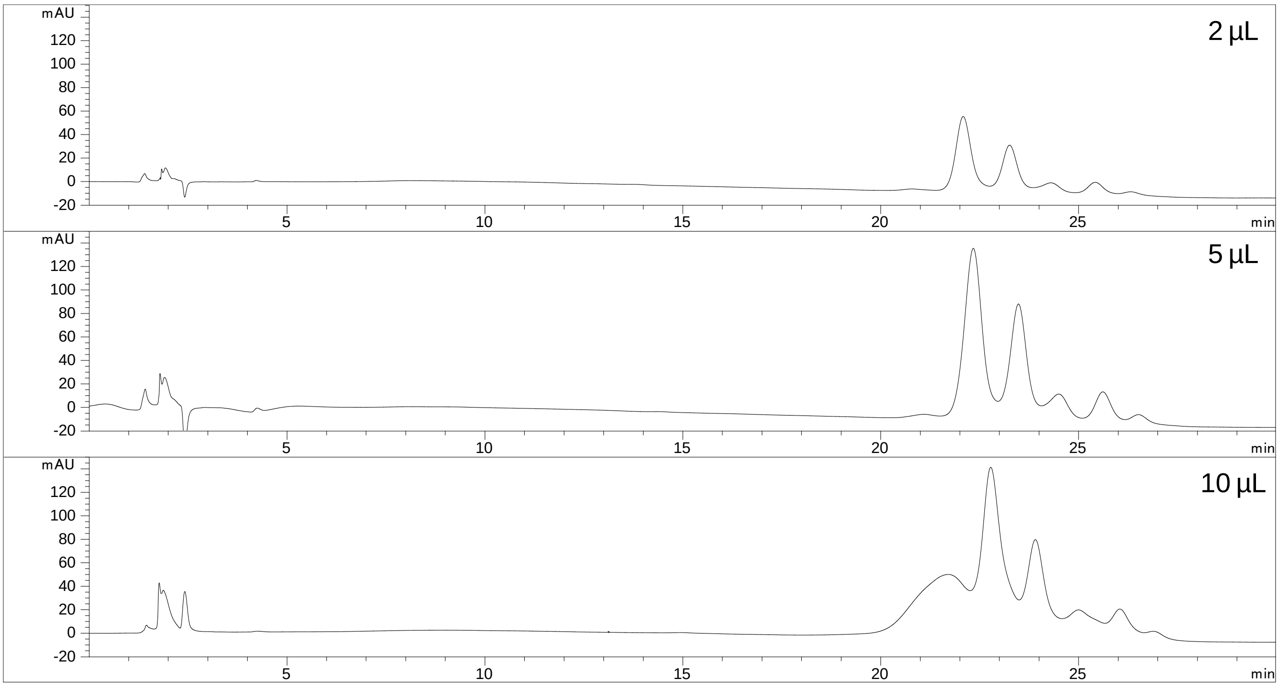

Figure S4. ESI-MS^2 and MS^3 spectra of ions *m/z* = 617.17 (Panel **A** and **B**) and *m/z* = 558.16 (Panel **C** and **D**).

A)

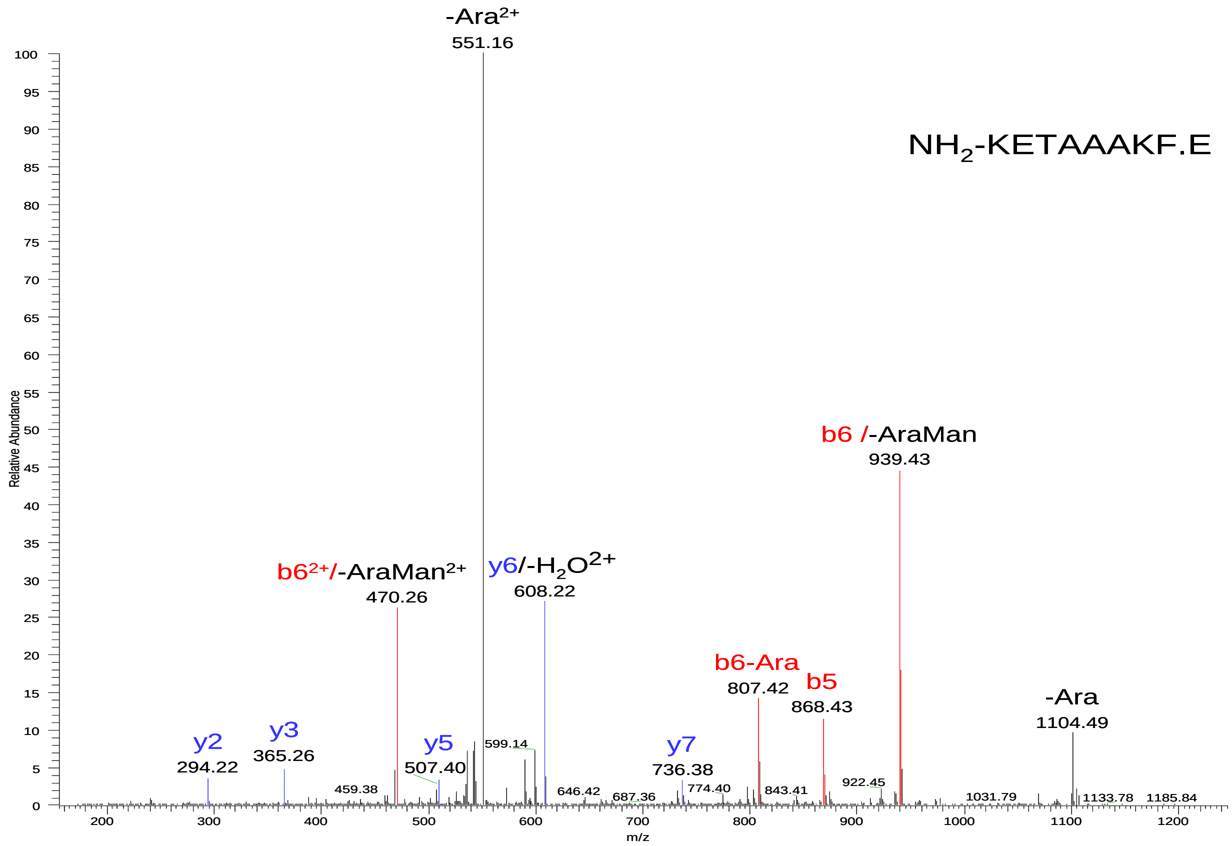

B)

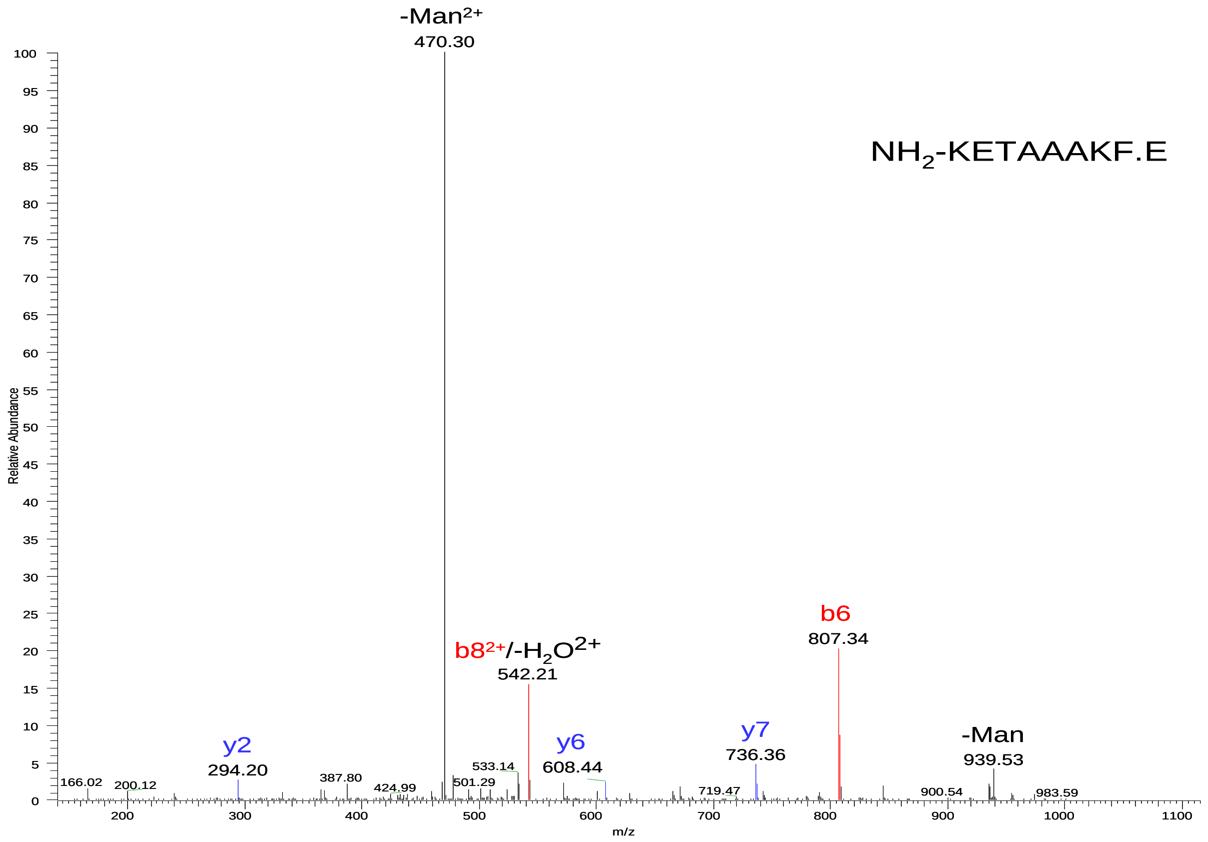

Figure S4. *Cont.*

C)

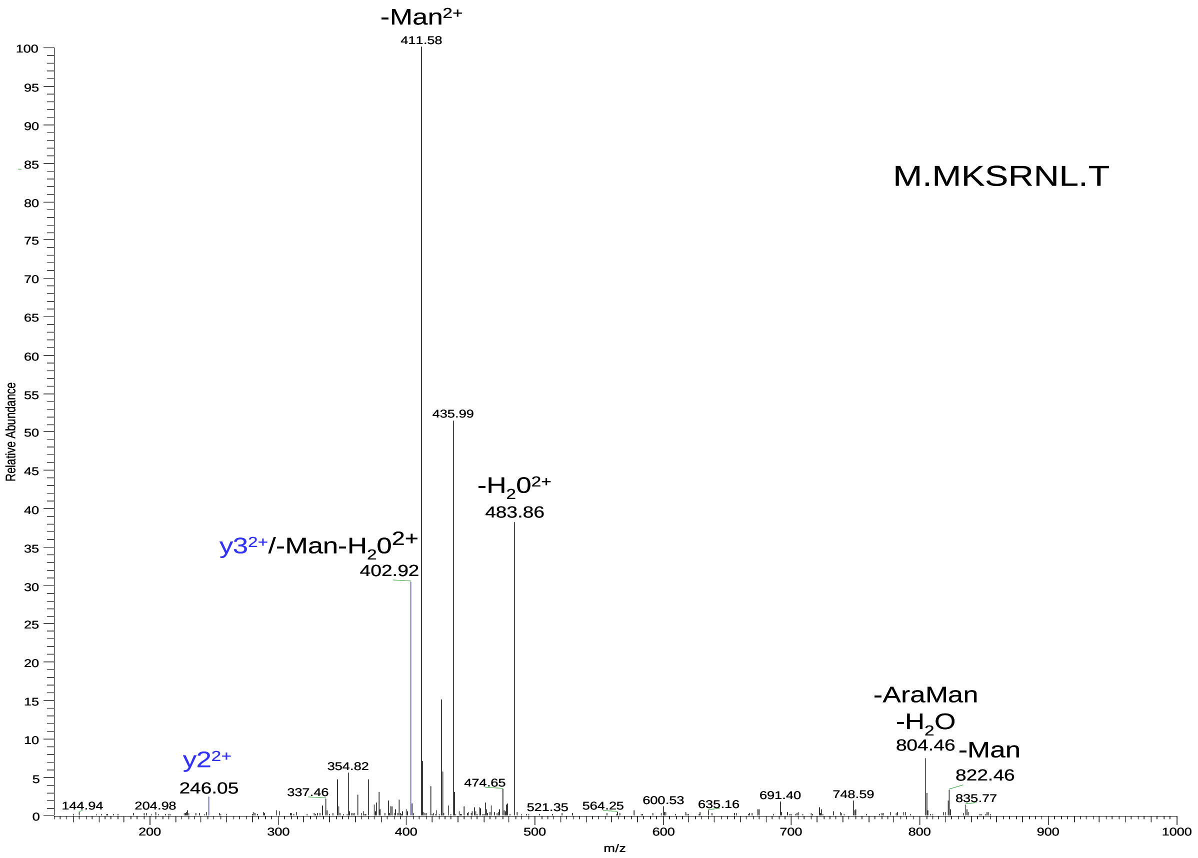

D)

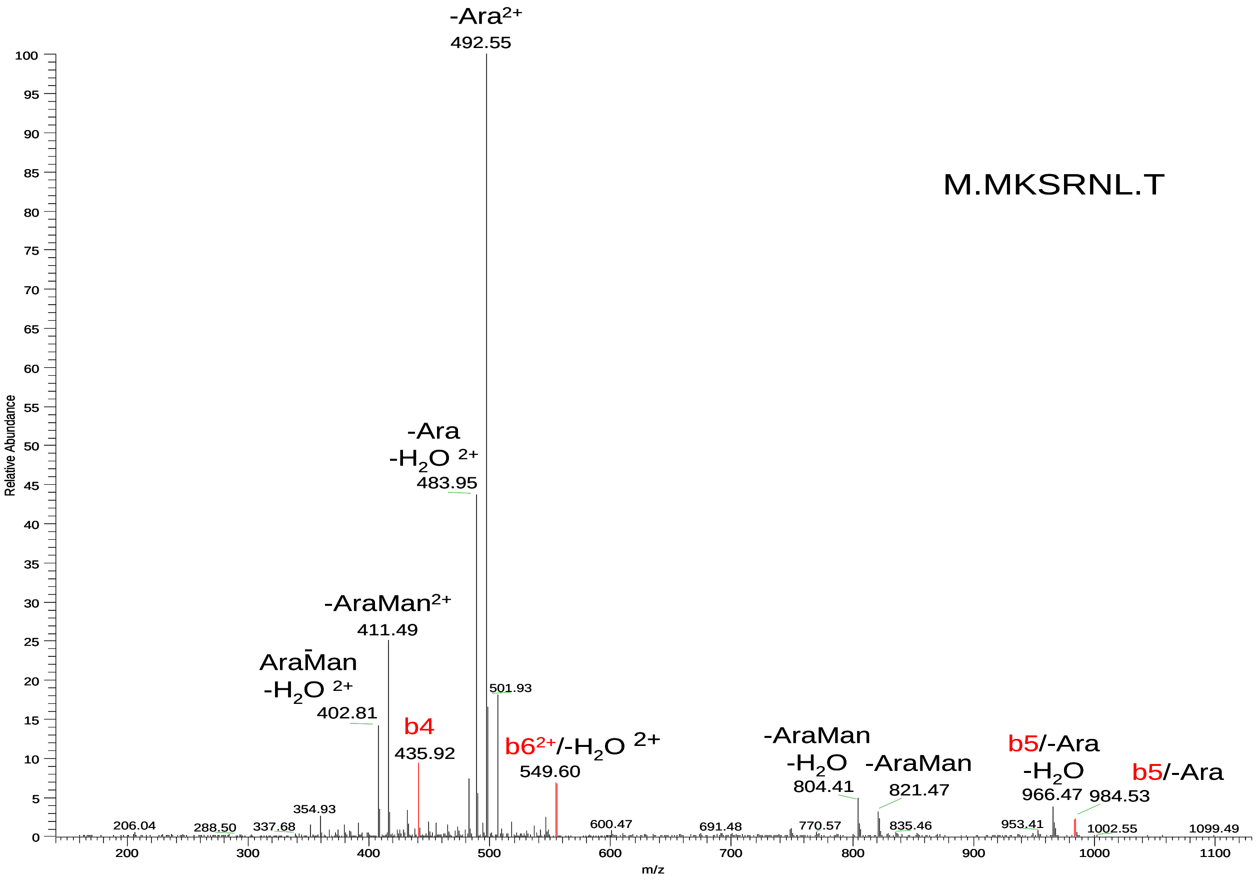

Supplement: Supplementary file 1 [file molecules-19-09070-s001.pdf]
